# *Clostridium* and *Cryptosporidium* outbreak linked to a splash pad

**DOI:** 10.1186/s12889-024-18870-7

**Published:** 2024-06-12

**Authors:** Anna de Andrés Aguayo, Joan-Pau Millet, Laia Álvarez-Bruned, David Palma, Anna Gómez, Pau Gallés, Sara Sabaté, Gabriela Álvarez, Virginia Rodriguez, Thais Cornejo, Cristina Rius

**Affiliations:** 1 Servei d’Epidemiología. Agència de Salut Pública de Barcelona, Plaza Lesseps 1, Barcelona, 08023 Spain; 2 Agència de Salut Pública de Barcelona, Plaza Lesseps 1, Barcelona, 08023 Spain; 3 Centro de Investigación Biomédica en Red de Epidemiología y Salud Pública, Plaza Lesseps 1, Barcelona, 08023 Spain; 4 Public Health Department of Reus City Council, Ample, Reus, 13–43202 Spain; 5 Vall d’Hebron Hospital Universitari, Plaza Lesseps 1, Barcelona, 08023 Spain

**Keywords:** Outbreak, AGE, *Cryptosporidium* spp., *Clostridium perfringens*, Splash pad, Spray park, Walkable fountains, Waterborne disease outbreak, Epidemiology, Epidemiological surveillance

## Abstract

**Background:**

. Splash pads for recreational purposes are widespread. Using these pads can pose a health risk if they lack installation regulation and water quality supervision. Our aim was to describe a waterborne disease outbreak caused by *Clostridium perfringens* and *Cryptosporidium* spp. in a Barcelona district and the measures taken for its control.

**Methods:**

. On August 2018, 71 cases of acute gastroenteritis were detected, affecting people who used a splash pad or were in contact with a user. Microbiological and environmental investigations were carried out. A descriptive analysis of the sample and Poisson regression models adjusted for age and sex were performed, obtaining frequencies, median values, and adjusted prevalence ratios with their 95% confidence intervals.

**Results:**

The median age of the cases was 6.7 years, 27 (38%) required medical care, and three (4.2%) were hospitalized. The greater the number of times a person entered the area, the greater the number of symptoms and their severity. Nineteen (76%) of the 25 stool samples collected from cases showed the presence of one or both pathogens. Environmental investigations showed deficiencies in the facilities and identified the presence of both species in the splash pad. Health education and hygiene measures were carried out, and 14 days after the closure of the facilities, no more cases related to the pad were recorded.

**Conclusions:**

. Specific regulations are needed on the use of splash pads for recreational purposes. Until these regulations are in place, these types of facility should comply with the regulations that apply to swimming pools and spas, including those related to the design of the tanks, water recirculation systems, and adequate disinfection systems.

## Introduction

In various cities in Europe and North America, the use of splash pads, areas where water jets are integrated into the ground surface, often with no standing water, is increasingly widespread, especially during summer, when people use them to cool off [1, 2]. Using such walkable fountains for this purpose may pose a health risk due to waterborne infections, especially if there are no regulations in place regarding the installation and supervision of these fountains [3]. In recent years, countries such as the United States [3–10], United Kingdom [11], the Netherlands [12] and Belgium [12], among others, have described outbreaks of infectious diseases in this type of facility as well as in spas, swimming pools, lakes, and water parks. The main pathogen described is *Cryptosporidium* spp., accounting for 19% of recreational water outbreaks between 2018 and 2019 in the US [10].

*Cryptosporidium* spp. is the second major cause of moderate to severe diarrhea in children younger than 2 years and is an important cause of mortality worldwide. Infection with these parasites most commonly occurs during waterborne epidemics and in immunocompromized hosts. Most episodes of cryptosporidiosis in immunocompetent hosts are self-limiting, which may lead to their undersuspicion and underdiagnosis. However, the infection may be associated with chronic symptoms, malnutrition, and other complications in high-risk patients [13].

The burden of cryptosporidiosis in Europe is difficult to estimate due to the lack of standardized surveillance and monitoring systems. Nevertheless, the increasing incidence of food and waterborne outbreaks suggests that *Cryptosporidium* spp. could be widespread in Europe [14]. Previous studies have reported a prevalence of *Cryptosporidium* spp. of 18.8% in pools in Barcelona [15], 16.6 in pools in Paris [16], and 28.5% in Palermo [17]. In Spain, where cryptosporidiosis requires mandatory reporting, previous outbreaks have been related to swimming pools and tap water [18–21]. In addition, other pathogens, such as *Clostridium perfringens*, mainly linked to foodborne outbreaks, could also play a role in causing acute gastroenteritis (AGE) outbreaks in these facilities [15, 22].

The aim of this study was to describe the investigation of an AGE outbreak in a splash pad in the city of Barcelona and the measures taken for its control.

## Methods

### Outbreak detection

On August 30, 2018, the Epidemiology Department of the Public Health Agency of Barcelona received an email from a mother whose child had played in a splash pad in the Sant Andreu district of Barcelona, an area with a socioeconomic indicator slightly below the average for the city [23]. She explained that her child had AGE and cutaneous symptoms and knew of other users with similar symptoms after cooling off in the facilities. The nursing team telephoned the mother and asked her to share their telephone number with parents whose children had played in the same splash pad and also had a history of AGE symptoms. Numerous calls were received, and within 24 h, 37 cases were recorded.

### Epidemiological investigations

A cross-sectional study was conducted by the Epidemiology Department to identify both primary and secondary cases. Subsequently, team members inspected the interactive fountain area and made the decision to order its temporarily closure.

### Case definition

People were considered primary cases if they had entered the splash pad area and had either gastrointestinal symptoms compatible with *C. perfringens* infection (diarrhea and abdominal pain) in the following 24 h, or intestinal symptoms compatible with *Cryptosporidium* spp. infection (diarrhea, abdominal pain, fever, nausea, and vomiting) in the following 1–12 days. Secondary cases were considered those without prior use of the splash pad who, after being in contact with a symptomatic case, developed symptoms of gastroenteritis compatible with both pathogens in the following 1–12 days. The study population were the people who cooled off in the interactive fountain between its opening on August 10, 2018, and its closure on August 30, 2018, and who had AGE symptoms.

### Data collection

A specific questionnaire was designed for the outbreak and was administered by telephone interview to all suspected primary and secondary cases. The questionnaire recorded age, sex, date of exposure to the fountain area, date of symptom onset, symptom duration and characteristics, need for medical care (primary or hospital care), illness prior to cooling off in the area, contact with sick people prior to using the fountains, and the presence of other people in their environment who later developed symptoms.

At the beginning of the epidemiological investigation, information was collected on food and visits to restaurants to rule out a foodborne outbreak. Information related to the cases was obtained from the outbreak record and the epidemiological surveys. The data were completed using the Clinical Health Shared Record of Catalonia.

Due to the lack of a record of users of the facilities, in an initial phase of the investigation, cases were detected through word of mouth among residents of the area through social media (WhatsApp, Facebook and Twitter), with the collaboration of the first primary case. This, together with dissemination in the local press and television, allowed identification and recording of a larger number of affected people. Once the cause of the outbreak was determined, to reach the maximum number affected users of the facilities, a list of all fecal isolates of *Cryptosporidium* spp. in August and September was requested from reference laboratories in the same area as the fountain. To determine whether there was a history of using the fountains, cases with positive *Cryptosporidium* spp. samples were contacted if they had had gastrointestinal symptoms after inauguration of the splash pad and had no clear epidemiological cause that could explain the infection. Because the data used for this study were drawn from epidemiological surveillance, retrieved, anonymized and stored under Spanish legislation [24], there was no mandatory requirement for its approval by an ethics committee.

### Clinical microbiological investigations

Stool samples were requested from the cases that remained symptomatic after identification of the outbreak. All samples were sent to the Laboratory of the Public Health Agency of Barcelona. Stool analysis included detection of *Salmonella* spp., *Campylobacter* spp., *Escherichia coli* O157, norovirus genogroups I and II, type A enterotoxin and spore count for *C. perfringens*, and coagulase-positive staphylococci. Subsequently, the same stool samples were sent to the reference laboratory for outbreaks in Catalonia (Microbiology Service of the University Hospital Vall d’Hebron) for microscopy analysis of parasites, which included *Cryptosporidium* spp. and *Giardia* spp.

### Environmental investigations

The day the outbreak was declared (August 30, 2018), the splash pad, consisting of 234 water jets, was inspected. It consists of 13 water lines with jets. Each line has its own vessel from which water is pumped to the jets. Ejected water is collected by gravity in a general tank, where it is filtered and disinfected with sodium hypochlorite. From there, water is recycled to the vessels. The fountains are situated in a permanently open urban area, with the possibility of animals or people wearing shoes passing through it.

The water was analyzed in situ to determine turbidity, free chlorine, and combined chlorine. The installation and the automatic chlorination system were checked. Four water samples were taken from the tank, the pumping system, the water jets, and the vessel drain on August 30, 2018, and September 3 and 14, 2018, and were sent to the laboratory of Aigües de Barcelona to check for *Giardia lamblia* and *Cryptosporidium* spp. The samples were also sent to the Laboratory of the Public Health Agency of Barcelona for pathogens and indicators of fecal contamination.

### Statistical analysis

We performed a descriptive analysis of the cases and the course of the outbreak. To evaluate the impact of the number of visits to the splash pad, we performed an exploratory Poisson regression, adjusting for age and sex in a single model and obtaining adjusted prevalence ratios (APR) with their 95% confidence intervals (95%CI). All analyses were conducted using STATA 15 software.

## Results

### Epidemiological investigations

A total of 80 epidemiological surveys were conducted between August 30th and September 19th; during that period, 71 people met the case definition. Among the 71 cases, 39 (54.9%) were women, and the median age was 6.7 (interquartile range (IQR): 3.4–20.7) years. The average incubation period was 2 (IQR: 1–8) days and the median symptom duration was 4.5 (IQR: 2–7) days. All cases had some AGE symptoms. The main symptoms were diarrhea (97.2%), abdominal pain (71.8%), nausea (29.6%), vomiting (29.6%), and fever (19.7%). Twenty-seven cases (38%) required medical care and 3 cases (4.2%) required hospital admission. A total of 61 (85.9%) primary cases and 10 (14.1%) secondary cases were recorded that were compatible with *Cryptosporidium* spp. infection (Table 1). Primary cases included confirmed cases with positive results for *Cryptosporidium* spp. or/and *Clostridium perfringens* or/and *G. lamblia* in feces, and 42 untested cases without a sample. Secondary cases refer to cases without a history of splash pad, who were in contact with primary cases.

**Table 1:** Descriptive Analysis of Confirmed Cases of Acute Gastroenteritis and Fecal Pathogens from August 10, 2018, to September 15, 2018

Variable	Median	Interquartile range (IQR)
Age (years)	6.7	3.4–20.7
Incubation period (days)	2	1–8
Duration of symptoms (days)	4.5	2–7
**Variable**	**N**	**%**
Sex		
Female	39	54.9%
Male	32	45.1%
AGE symptoms (multiple option)		
Diarrhea	69	97.2%
Abdominal pain	51	71.8%
Nausea	21	29.6%
Vomiting	21	29.6%
Fever	14	19.7%
Not specified	2	2.8%
Non-AGE symptoms (multiple option)		
Cutaneous symptoms	13	18.3%
Eye irritation	2	2.8%
Complications		
Medical consultation	27	38.0%
Hospitalization	3	4.2%
Classification of case		
Primary cases	61	85.9%
Secondary Cases	10	14.1%
Number of visits to the splash pad per case		
None	12	16.9%
One	18	25.3%
Two	21	29.6%
Three	10	14.1%
Four or more	10	14.1%
Samples collected	25	35.2%
Positive samples	19	26.8%
*Cryptosporidium spp*.	7	9.9%
*Clostridium perfringens*	2	2.8%
*C. perfringens* + *Cryptosporidium*	9	12.7%
*Cryptosporidium* + *Giardia lamblia*	1	1.4%

**Fig. 1:**
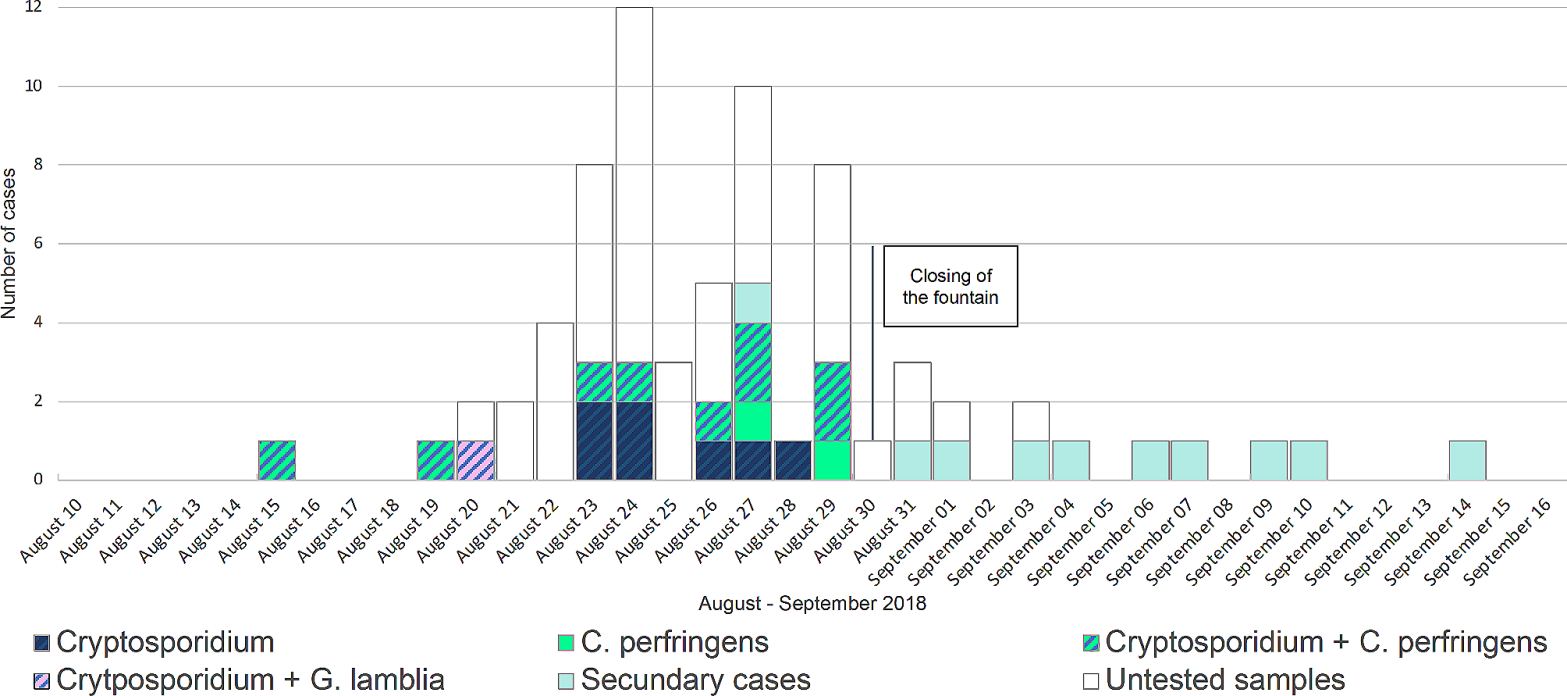
Epidemic curve of the cases of acute gastroenteritis and pathogens in feces from August 10, 2018, until September 15, 2018. C. perfringens: *Clostridium perfringens*. G. lamblia: *Giardia lamblia*

The first case developed symptoms 6 days after inauguration of the splash pad. The epidemic curve of the outbreak extended from August 15 to September 14, 2018, 14 days after closure of the fountain area. The last primary case developed symptoms 4 days after closure of the fountains. Subsequently, all secondary cases (with symptoms compatible with cryptosporidiosis) were cohabitants of primary cases, suggests that entering the fountain area was the common point of infection (Fig. 1).

Poisson analysis showed an association between a larger number of times a person entered the area and the presence of cutaneous symptoms (APR: 1.54; 95%CI: 1.06–2.25) and requiring hospitalization (APR: 2.01; 95%CI: 1.09–3.73), with a weak association with younger age (APR: 0.97; 95%CI: 0.96–0.99) (Table 2):

**Table 2:** Crude and adjusted Poisson regression of number of visits to the splash pad

Variable	CPR	95%CI	APR	95%CI
Sex (female)	1.30	0.93–1.82	1.35	0.96–1.89
Age	0.98	0.96–0.98	0.97	0.96–0.99
Cutaneous symptoms	1.75	1.23–2.52	1.54	1.06–2.25
Fever	1.61	1.12–2.30		
Nausea	1.54	1.10–2.15		
Vomiting	1.37	0.98–1.92		
Contact with case	0.63	0.43–0.92		
Hospitalization	2.05	1.13–3.69	2.01	1.09–3.73
Medical consultation	1.06	0.76–1.47		
Duration of symptoms	0.96	0.91–1.01		

CPR: crude prevalence ratio. APR: adjusted prevalence ratio, including all variables presented in a single model. Sex variable: female versus male. Age is presented as a numeric variable. Symptom duration in days

### Microbiological investigations

Stool samples were collected from 25 (35.2%) cases, of which 19 (76%) were positive: nine (36%) simultaneously showed the presence of *Cryptosporidium* spp. and *C. perfringens*, 7 (28%) were positive only for *Cryptosporidium* spp., two (8%) were positive only for *C. perfringens*, and 1 (4%) was positive for both *Cryptosporidium* spp. and *Giardia lamblia* (Table 1).

### Environmental investigations

In the first 2 health inspections (August 30, 2018 and September 3, 2018), several breaches of public swimming pool regulations were detected. Likewise, water treatment was found to be inadequate. Daily records of the facility showed a lack of compliance with water turbidity requirements (> 5 NTU) and combined free chlorine values (> 0.6 ppm). The automatic chlorination system was found not to work properly.

Analysis of the water samples, collected from the fountain on August 30, 2018, showed the presence of aerobic microorganisms (> 3,000 CFU/L) in all samples, coliform bacteria in 3 of the 4 samples, and *C. perfringens* in 2 of the 4 samples. These samples also showed the presence of *Cryptosporidium* spp. and *G. lamblia*. In the water samples collected on September 3 and 14, 2018, after cleaning and disinfection, no presence of *C. perfringens* or *Cryptosporidium* spp. was detected.

### Outbreak control measures

After the outbreak was declared on August 30, 2018, the facilities were closed as a preventive measure. In addition, cleansing and disinfection treatments, including super chlorination, were performed. Information was provided to all infected individuals regarding standard hygiene precautions to avoid new cases appearing in their homes and in the community. Children whose fecal samples were positive for *Cryptosporidium* spp. were advised not to attend school or use water facilities for at least 15 days after symptom onset. The primary care centers in the area were contacted to inform them about the outbreak and request their collaboration in detecting new cases, especially those geographically closer to the fountain. Pediatricians specialized in infectious diseases collaborated by providing a reference for the management of cases in primary care, especially those requiring treatment.

## Discussion

The data suggest that the route of transmission of the outbreak was water from an interactive fountain, between August 10 and 30, 2018, and the infectious agents that caused it were *Cryptosporidium* spp. and *C. perfringens*. Environmental investigations were consistent with epidemiological findings and revealed severe deficiencies in the design and maintenance of the splash pad. Both pathogens were identified in water samples collected from different points of the facilities, and in the samples from people who had gastroenteritis. *Cryptosporidium* spp. was found in 68% of the fecal samples and *C. perfringens* in 44%; both pathogens together were found in 36% of the samples. Closure of the facilities, following the declaration of the outbreak on August 30, ended the emergence of new primary cases after September 3, 2018.

The observed results are consistent with outbreaks with similar characteristics described in this type of recreational area in other countries, with reports of pathogens such as *Giardia* spp., *Shigella* spp., and norovirus [12, 25–27]. However, there is no literature on similar outbreaks in this type of facility where the disease was caused by *C. perfringens*. This pathogen usually causes food poisoning, although it has less frequently been related to waterborne outbreaks [22]. Of note, *C. perfringens* may have played a substantial role in the development or exacerbation of gastrointestinal symptoms, especially in persons affected by both pathogens. Our analyses found associations between a greater number of visits to the splash pad, younger age, and an increased risk of hospitalization and cutaneous symptoms, supporting a causal association consistent with dose-response exposure. Similar analyses in Maine (2018) found that individuals who swallowed pond water or immersed themselves under water, were approximately 3 times more likely to become ill than those who did not [10].

Measures to control and prevent transmission of enteric pathogens through untreated recreational water include epidemiologic investigations, regular monitoring of water quality, microbial source tracking, and health policy and communications. Investigations include health inspection of the septic system, identification of agricultural animal waste runoff or discharge, monitoring wildlife activity in public areas, and the identification of improper disposal of solid waste [10].

In Spain, Royal Decree 742/2013 [28] sets out specific requirements regarding microbiological criteria and swimming pool monitoring: for every 100 mL of pool water analyzed, no *E. coli* or *Pseudomonas aeruginosa* should be detected. In addition, *Legionella* spp. monitoring is mandatory in heated pools or pools with aeration in the pool vessel, and concentrations must be lower than 100 CFU/L [15]. However, due to the lack of specific regulation regarding the use of splash pads, we believe that their design and construction, as well as the requirements for their maintenance, do not fit with their real use. For this reason and the increasing installation of these types of facilities as climate shelters in cities [29, 30], we believe it is essential that the relevant authorities approve a specific regulation regarding these types of fountains. The design of the installation analyzed in this study only included disinfection with sodium hypochlorite, and the chlorine levels detected varied among the different points of the installation. Furthermore, *Cryptosporidium* has been associated with swimming pool outbreaks due to its strong resistance to chlorine and resistance to elimination by filtration [31]. Other countries with more experience in the use of this type of facility have guidelines that recommend the use of ultraviolet light (in addition to chlorination) for water disinfection, since this method has proven to be more effective in eliminating cyst-forming pathogens such as *Cryptosporidium* spp [32].

Enteric pathogens can be transmitted when individuals ingest untreated recreational water contaminated with feces or vomit introduced in water by other swimmers or by storm water runoff and sewage system overflow and discharge, as well as leaks from septic or municipal wastewater system, dumped boating waste, and animal taste in or near swimming areas [10]. The installation studied here was at high risk of microbiological contamination, since its area was not closed to prevent the transit of users with shoes or the entry of animals. Additionally, the users of these facilities are usually young children, increasing the probability of fecal contamination of water, due to the use of diapers and a greater degree of incontinence. These circumstances should be corrected by closing the perimeter of the facilities and recommending adequate hygiene measures prior to their use (e.g., use of showers, absence of footwear, appropriate clothing). We believe these recommendations should be included in national guidelines, as in other countries such as the Netherlands and Canada [32–34].

The main limitation of this study is that, due to the lack of records on the people visiting the facilities during the days it remained in operation, we were unable to estimate the total number of people who became ill. For this reason, and because active case detection was only carried out in the city of Barcelona, it is highly likely that not all cases of infection after splash pad use were detected. Given that several cases had used the facilities repeatedly, the incubation period could not be accurately calculated and, consequently, the last date of splash pad use was recorded as the exposure date.

In contrast, a strength of this study was the dissemination of information through social media, which allowed information to be collected from a large number of affected individuals.

## Conclusions

The use of splash pads without appropriate recirculation and disinfection systems can put human health at risk for waterborne diseases. To date, Spain lacks a specific regulation on these facilities. Areas designed for recreational water use and cooling off should comply with the regulations that apply to swimming pools and spas, taking into account the design of the tanks, water recirculation systems, and adequate disinfection systems. Given the climate emergency, which will lead to an increase in the abovementioned facilities and climate shelters, urgent action is needed.

Prior to the opening of more interactive fountain areas with these characteristics, public health authorities should be involved in verifying compliance with the necessary requirements to ensure the safety of the population.

## Data Availability

The datasets generated and analyzed during the current study are not publicly available due to confidentiality reasons but are available from the corresponding author on reasonable request.

## Abbreviations

AGE: Acute Gastroenteritis
APR: Adjusted Prevalence Ratios
CFU/L: Colony-forming Unit per Liter
95%CI: 95% Confidence Interval
IQR: Interquartile Range
NTU: Nephelometric Turbidity Unit
PPM: Parts per Million

## References

[1] Hlavsa MC, Roberts VA, Anderson AR, Hill VR, Kahler AM, Orr M et al. Surveillance for waterborne disease outbreaks and other health events associated with recreational water - United States, 2007–2008. MMWR Surveill Summ [Internet]. 2011;60(12):1–32. http://www.ncbi.nlm.nih.gov/pubmed/21937976. 21937976

[2] Centers for Disease Control and Prevention. Swimming-associated cryptosporidiosis -- Los Angeles County [Internet]. Epidemiologic notes and reports. 1990. pp. 343–5. https://www.cdc.gov/mmwr/preview/mmwrhtml/00001630.htm. Accessed 14 Dec 2022.

[3] Ryan U, Lawler S, Reid S (2017). Limiting swimming pool outbreaks of cryptosporidiosis, the roles of regulations, staff, patrons and research. J Water Health.

[4] Centers for Disease Control and Prevention (CDC) (1999). Outbreak of cryptosporidiosis associated with a water sprinkler fountain, Minnesota, 1997. Can Commun Dis Rep.

[5] Jue R, Schmalz T, Carter K, Nett R (2009). Outbreak of Cryptosporidiosis Associated with a Splash Park --- Idaho, 2007. MMWR Morb Mortal Wkly Rep.

[6] Hlavsa MC, Aluko SK, Miller AD, Person J, Gerdes ME, Lee S et al. Outbreaks Associated with Treated Recreational Water — United States, 2015–2019. MMWR Morb Mortal Wkly Rep [Internet]. 2021;70(20):733–8. http://www.cdc.gov/mmwr/volumes/70/wr/mm7020a1.htm?s_cid=mm7020a1_w. Accessed 14 Dec 2022. 10.15585/mmwr.mm7020a1 PMC8136425 34014907

[7] Centers for Disease Control and Prevention (CDC). No Germs Allowed: Tennessee FoodCORE Team Aims to Keep Splash Pads Safe and Fun [Internet]. 2015. https://www.cdc.gov/foodcore/successes/tennessee-splash-pad.html. Accessed 14 Dec 2022.

[8] Sorvillo FJ, Fujioka K, Nahlen B, Tormey MP, Kebabjian R, Mascola L (1992). Swimming-associated cryptosporidiosis. Am J Public Health.

[9] Cantey PT, Kurian AK, Jefferson D, Moerbe MM, Marshall K, Blankenship WR (2012). Outbreak of cryptosporidiosis associated with a man-made chlorinated lake-tarrant county, Texas, 2008. J Environ Health.

[10] Vanden Esschert KL, Mattioli MC, Hilborn ED, Roberts VA, Yu AT, Lamba K (2020). Outbreaks Associated with untreated recreational water - California, Maine, and Minnesota, 2018–2019. MMWR Morb Mortal Wkly Rep.

[11] Jones M, Boccia D, Kealy M, Salkin B, Ferrero A, Nichols G (2006). Cryptosporidium outbreak linked to interactive water feature, UK: importance of guidelines. Euro Surveill.

[12] Hoebe CJPA, Vennema H, De Roda Husman AM, Van Duynhoven YTHP (2004). Norovirus Outbreak among primary schoolchildren who had played in a recreational water fountain. J Infect Dis.

[13] Janssen B, Snowden J. Cryptosporidiosis. In: StatPearls [Internet]. Treasure Island (FL): StatPearls Publishing; 2022 Jan. Accessed 14 Dec 2022.

[14] Cacciò SM, Chalmers RM. Human cryptosporidiosis in Europe. Vol. 22, Clinical Microbiology and Infection. 2016. pp. 471–80. 10.1016/j.cmi.2016.04.021 27172805

[15] Gracenea M, Castaño S, Méndez J, Lucena F, Gómez MS (2018). Faecal contamination in public pools in Barcelona province: Cryptosporidium spp. and bacterial indicators. J Water Health.

[16] Fournier S, Dubrou S, Liguory O, Gaussin F, Santillana-Hayat M, Sarfati C (2002). Detection of microsporidia, cryptosporidia and giardia in swimming pools: a one-year prospective study. FEMS Immunol Med Microbiol.

[17] Oliveri R, Di Piazza F, Marsala B, Cerame G, Firenze A, Di Benedetto MA (2006). Occurrence of Giardia cysts and Cryptosporidium oocysts in swimming pools in the province of Palermo, Italy. Ann Ig.

[18] Rodríguez-Salinas Pérez E, Aragón Peña A-J, Allue Tango M, Lópaz Pérez MÁ, Jiménez Maldonado M (2000). Domínguez Rodríguez MJ. Brote De criptosporidiosis en Guadarrama (Comunidad Autónoma De Madrid). Rev Esp Salud Publica.

[19] Galmes A, Nicolau A, Arbona G, Smith-Palmer A, Hernández Pezzi G, Soler P. Cryptosporidiosis outbreak in British tourists who stayed at a hotel in Majorca, Spain. Wkly Releases. 2003;7(33).

[20] Artieda J, Basterrechea M, Arriola L, Yagüe M, Albisua E, Arostegui N (2012). Outbreak of cryptosporidiosis in a child day-care centre in Gipuzkoa, Spain, October to December 2011. Eurosurveillance.

[21] Fuentes I, Martín C, Beristain X, Mazón A, Saugar JM, Blanco A (2015). Cryptosporidium hominis genotypes involved in increased incidence and clusters of cases, Navarra, Spain, 2012. Epidemiol Infect.

[22] Dolan GP, Foster K, Lawler J, Amar C, Swift C, Aird H et al. An epidemiological review of gastrointestinal outbreaks associated with Clostridium perfringens, North East of England, 2012–2014. 144, Epidemiology and Infection. 2016. p. 1386–93. 10.1017/S0950268815002824 PMC9150519 26567801

[23] Ajuntament de Barcelona. Distribució Territorial de la Renda familiar disponible per càpita a Barcelona. 2018 [Internet]. 2021. https://ajuntament.barcelona.cat/barcelonaeconomia/ca/renda-familiar/renda-familiar/distribucio-territorial-de-la-renda-familiar-disponible-capita. Accessed 14 Dec 2022.

[24] Department of Health. Decree 203/2015, of September 15, establishing the Epidemiological Surveillance Network and regulating the notification systems for diseases of mandatory declaration and epidemic outbreaks. Barcelona: Parliament of Catalonia; Sep 15, 2015.

[25] Ehsan A, Casaert S, Levecke B, Van Rooy L, Pelicaen J, Smis A (2015). Cryptosporidium and Giardia in recreational water in Belgium. J Water Health.

[26] Fleming C, Caron D, Gunn J, Horine M, Matyas B, Barry M (2000). An outbreak of Shigella sonnei associated with a recreational spray fountain. Am J Public Health.

[27] Shields JM, Hill VR, Arrowood MJ, Beach MJ (2008). Inactivation of Cryptosporidium parvum under chlorinated recreational water conditions. J Water Health.

[28] Ministry of Health, Social Services, and Equality. Royal Decree 742/2013, of September 27, establishing the technical-health criteria for swimming pools. Spain. Official State Gazette (BOE) no. 244, dated October 11, 2013.

[29] Ajuntament de Barcelona. Climate shelters in schools [Internet]. 2018. https://www.barcelona.cat/barcelona-pel-clima/en/climate-shelters-schools. Accessed 14 Dec 2022.

[30] Cartalis C. The Climate Shelters project Journal N° 2 [Internet]. Barcelona; 2020. https://uia-initiative.eu/sites/default/files/2021-02/GBGAS2C_Barcelona_Journal2.pdf. Accessed 14 Dec 2022.

[31] Flugelman AA, Dubnov J, Jacob L, Stein N, Habib S, Rishpon S (2019). Epidemiologic Surveillance in Israel of Cryptosporidium, a Unique Waterborne Notifiable Pathogen, and Public Health Policy. Isr Med Assoc J.

[32] Russell C, Eykelbosh A. Identifying and Addressing the Public Health Risks of Splash Parks Key Messages [Internet]. 2017. https://ncceh.ca/documents/evidence-review/identifying-and-addressing-public-health-risks-splash-parks. Accessed 14 Dec 2022.

[33] De Man H, Leenen EJTM, Van Knapen F, De Roda Husman AM (2014). Risk factors and monitoring for water quality to determine best management practices for splash parks. J Water Health.

[34] Centers for Disease Control and Prevention (CDC). Splash pads [Internet]. 2022. https://www.cdc.gov/healthywater/swimming/swimmers/water-play-areas-interactive-fountains.html. Accessed 14 Dec 2022.

